# Assessment of ipsilateral and contralateral perfusion after contrast compression therapy of upper limb muscles in MMA athletes - a cross-over study

**DOI:** 10.3389/fphys.2024.1498590

**Published:** 2024-12-18

**Authors:** Robert Trybulski, Arkadiusz Stanula, Jarosław Muracki, Wacław Kuczmik, Ahmet Kurtoğlu, Jakub Taradaj

**Affiliations:** ^1^ Provita Żory Medical Center, Żory, Poland; ^2^ Medical Department, The Wojciech Korfanty Upper Silesian Academy, Katowice, Poland; ^3^ Institute of Sport Science, The Jerzy Kukuczka Academy of Physical Education in Katowice, Katowice, Poland; ^4^ Institute of Physical Culture Sciences, Department of Physical Culture and Health, University of Szczecin, Szczecin, Poland; ^5^ Department and Clinic of General Surgery, Vascular Surgery, Angiology and Phlebology, Uppersilesian Medical Center, Medical University of Silesia, Katowice, Poland; ^6^ Department of Coaching Education, Faculty of Sport Science, Bandirma Onyedi Eylul University, Balikesir, Türkiye; ^7^ Institute of Physiotherapy and Health Sciences, The Jerzy Kukuczka Academy of Physical Education in Katowice, Katowice, Poland

**Keywords:** microcirculation, game-ready therapy, cross-effect, combat sport, recovery

## Abstract

**Objective:**

The primary aim of this study was to compare the immediate effect of contrast compression therapy with the use of Game Ready (GRT) on hyperaemic reactions in the upper limb on the application and contralateral sides, specifically in the context of mixed martial arts (MMA) athletes.

**Design:**

In this experimental, single-blind, randomized crossover study, we recruited 30 male volunteers training in MMA (mean age: 28.33 ± 3.79 years, BMI: 25.25 ± 3.06, training experience: 9.93 ± 3.83). They were randomly assigned to the experimental (n = 15) or control (sham) group (n = 15). The experimental group underwent a 10-minute Game Ready Therapy (GRT) session, while the control group GRS underwent a sham therapy session. After a 2-week break, a cross-over change of therapy in the groups was performed, ensuring a comprehensive evaluation of the contrast compression therapy’s perfusion effects in 30 participants. *Main outcome measures:* Hyperemic reaction was measured: rest flow (RF - [non-referent unit]); therapeutic flow (TF- [min]), i.e., the average flow recorded during GR or sham therapy: time of recovery (TR - [min]), i.e., the time for perfusion to return to the resting value after the intervention. Measurements were performed on the ipsilateral and contralateral sides.

**Results:**

The mean perfusion during therapy was significantly higher in GRT compared to GRS (24.70 ± 1.45 vs. 12.60 ± 1.37; *p* < 0.001; ES = 5.7 [large]; △ = 12.10 > MDC). The time from cessation of contrast therapy to the return of blood flow to resting values showed significantly higher values in GRT compared to GRS (3.07 ± 0.45 vs. 16.80 ± 0.91; *p* < 0.001; ES = 16.27 [large]). No statistically significant difference was noted between the mean resting perfusion value (RF) and the mean perfusion value during therapy (TF) in the contralateral limb (7.74 ± 0.89 vs. 7.66 ± 0.89; *p* = 0.284; ES = 0.20 [negligible]; △ = 0.09 < MDC.

**Conclusion:**

This study suggests that compression contrast therapy on the ipsilateral side positively affects the intensification of the hyperaemic reaction. However, no statistically significant hyperaemic responses were observed on the contralateral side.

## 1 Introduction

Contrast therapy is a popular method used in clinical practice and sports training (Nahon et al., 2021), including MMA mixed martial arts training (Trybulski et al., 2024b). Several forms of contrast therapy can cover the whole body or part of it (Cochrane, 2004), including immersion in hot and cold water (CWT) (Versey et al., 2012) and combining microwave or ultrasonic diathermy with cryotherapy using innovative devices (McGorm et al., 2018). In the last few years, we have observed an increase in the popularity of an innovative contrast therapy strategy, described as Game Ready therapy (GR) (Diouf et al., 2018; Alexander et al., 2021), which belongs to the group of mechanoreceptive methods (Hawkins et al., 2012) and physical methods (Trybulski et al., 2024a). GR combines alternating hot and cold stimuli to tissue as a pressure cuff. The pressure we can use is variable and ranges from 15 to 75 mmHg, and the temperature from 3 to 45°C. The procedure lasts 10–30 min (Trybulski et al., 2024c). This therapy combines multiple stimuli: heat, cold, and compression.

One of the most crucial physiological variables for human health that the authors focus on is the changes in blood flow in microcirculation (Eriksson et al., 1986; Selkow et al., 2013). The role of microcirculation extends beyond mere blood flow regulation. Endothelial dysfunction is a critical phenomenon in the pathogenesis of systemic disease circulation and precedes structural changes in the vessels and clinical symptoms (Kvandal et al., 2006). It has a documented impact on cardiovascular diseases (Sirufo et al., 2021), muscle stiffness and tension (Kellogg, 2006; Goswami et al., 2022), injury prevention (Bahr and Holme, 2003), improvement of athletes’ performance (Szyguła et al., 2020), muscle strength (Hendrickse and Degens, 2019) anti-inflammatory effect (Zebrowska et al., 2019), anti-swelling effect (Diouf et al., 2018), and elimination of muscle fatigue (Hohenauer et al., 2018; Trybulski et al., 2024a). The availability of modern tools for assessing microcirculation, with laser Doppler flowmetry LDF being the most popular method due to its ease of measurement and documented repeatability (Roberts et al., 2017), has opened up new avenues for research in the field of sports science.

As part of these studies, the contralateral effect is also analyzed, known as the cross effect, which means that reactions and changes on the opposite side are observed due to different stimuli applied to the area of a given tissue (Shenker et al., 2003). The mechanisms underlying this cross-over effect have yet to be studied in detail. Still, most studies suggest that the nervous system mediates it (Lima et al., 2021) thanks to its unlimited possibilities related to plasticity (Hortobagyi et al., 2011). Previous research has confirmed the cross-effect in changes such as inflammation (Shenker et al., 2003), changes in muscle tone after massage (Lawrence et al., 2021; Miller et al., 2018), strength gains in response to exercise (Lee and Carroll, 2007) as well as electrical stimulation (Song et al., 2012), increase in hyperemia after acupuncture (Guangjun et al., 2012) regenerative reactions (Shenker et al., 2003), pain reduction (Colantuono et al., 2023) effect after blood flow resistance (BFR) training (Wong et al., 2024). There is evidence that a single bout of foam rolling (FR) on the ipsilateral limb increases the contralateral limb’s range of motion (ROM); however, evidence regarding long-term effects is limited. The most likely mechanism for increased contralateral ROM is decreased pain perception (Konrad et al., 2023). In general, changes in the contralateral limb are homologous and dependent on the type and strength of the stimulus, and the effect on the measured result is almost always smaller than on the ipsilateral side (Miller et al., 2018; Shenker et al., 2003).

Although there is some evidence for the effectiveness of GRT in reducing muscle fatigue (Alexander et al., 2021), relieving pain (Diouf et al., 2018), swelling (Cochrane, 2004), improving congestion (Trybulski et al., 2024a), and affecting changes in stiffness, elasticity, and muscle tone (Sawada et al., 2022; Trybulski et al., 2024a), there are several gaps in the scientific literature regarding this form of therapy. Previous studies suggest the beneficial effects of GRT in as little as 10 min of treatment (Trybulski et al., 2024a). However, no specific protocols exist for this therapy in clinical practice (Hawkins et al., 2012). There is no evidence regarding possible contralateral perfusion effects in GR therapy.

Contralateral effects are commonly used in physiotherapy and medicine practice. Therefore, this study aimed to assess the impact of GRT on perfusion of the forearm muscles of MMA training athletes and the effect of hyperemia on the contralateral side, which was not subjected to the intervention. MMA fighters are a very demanding group in terms of effective regeneration methods – their efforts are very intense, and often the amount of time for recovery is low so they need effective and time saving methods. Moreover, in the combat sport fighting and training different injuries can occur, including wounds and contusions which eliminates the possibility of usage of physical therapies on this sites. Considering this, the physicians may want to use the contralateral effect of the therapy to evoke hyperemia and increase the healing process of wounds and contusions. Analyzing the scientific literature, we concluded that our study assessing microcirculatory reactions on the contralateral side is innovative, and its results should be used to further develop research on contralateral effects in physiotherapy and medicine.

## 2 Materials and methods

### 2.1 Study design

**FIGURE 1:**
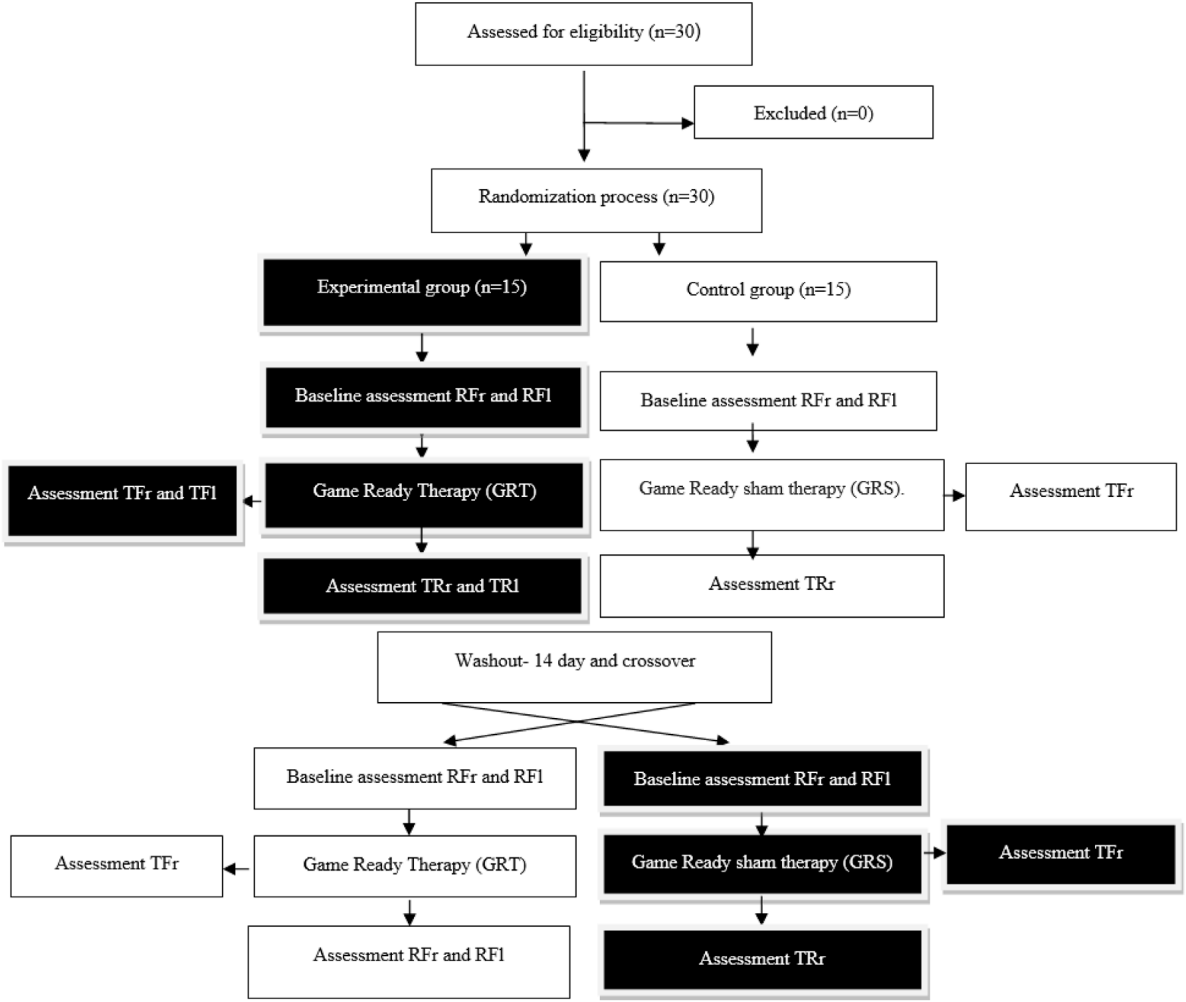
Study design. Experimental group - GRT–Game Ready Therapy group, control group - GRS–Game Ready sham therapy group, RFr and RFl–Resting Flow of right/left forearm, TFr and TFl–Therapeutic Flow of right/left forearm, TRr and TRl–Time Recovery of right/left forearm.

This study was a randomized crossover design. Participants were assigned to an experimental group (Game Ready Therapy – GRT, n = 15) and a control group (Game Ready Sham Therapy - GRS, n = 15). The experimental group received Game Ready contrast therapy for 10 min, and the control group received sham therapy. After a 2-week break, a crossover intervention was performed, which mean, that to the final data analysis there were 30 participants in the GRT and 30 participants in the GRS groups included. The study design is shown in Figure 1. Group allocation was achieved by simple 1:1 randomization using a random sequence generated on the randomizer.org website. The randomization process was independent of treatment duration and study personnel. The study was approved by the ethical committee of the National Council of Physiotherapists (no. 9/22 of 6 April 2022) and registered in the clinical trials register under the number ISRCTN90040217 and conducted by the Declaration of Helsinki.

### 2.2 Participants

Thirty male, young, healthy volunteers practicing MMA (age: 28.33 ± 3.79 years, BMI: 25.25 ± 3.06, training experience: 9.93 ± 3.83) (Table 1) were randomly selected according to the following criteria: age 18–35 years, minimum 3 years of experience in training martial arts, training at least four times in a week, taking into account McKay’s participant classification scheme, the group of fighters belonged to Level 3: Highly Trained/National level (McKay et al., 2022). The exclusion criteria were: blood pressure >140/90 mmHg; currently treated injuries, skin lesions, or unspecified skin lesions at the measurement sites; a tattoo at the measurement site, (as it makes it difficult to measure tissue blood supply) taking any medications, including painkillers. Exclusions were also made in the event of extreme fatigue, fever, infection, or at the explicit request of the participant (Holwerda et al., 2013). Before the study, participants were required to refrain from exercising for 24 h and from consuming any ergogenic drinks (they were provided with a list of excluded products) 24 h before the study (Trybulski et al., 2024c). Exclusion from the study could occur at any time during the study at the participant’s request. Before the study, each participant completed a health survey and gave informed consent to participate in the study.

**TABLE 1:** Essential characteristics of study group (n = 30).

Variables	Mean ± SD	Range
Age (year)	28.33 ± 3.79	24.0–36.0
Height (cm)	180.23 ± 7.61	156.0–197.0
Weight (kg)	81.00 ± 10.16	56.3–98.8
BMI (kg/m^2^)	25.25 ± 3.06	21.1–32.9
Training exper. (year)	9.93 ± 3.83	5.0–20.0

### 2.3 Interventions

**FIGURE 2:**
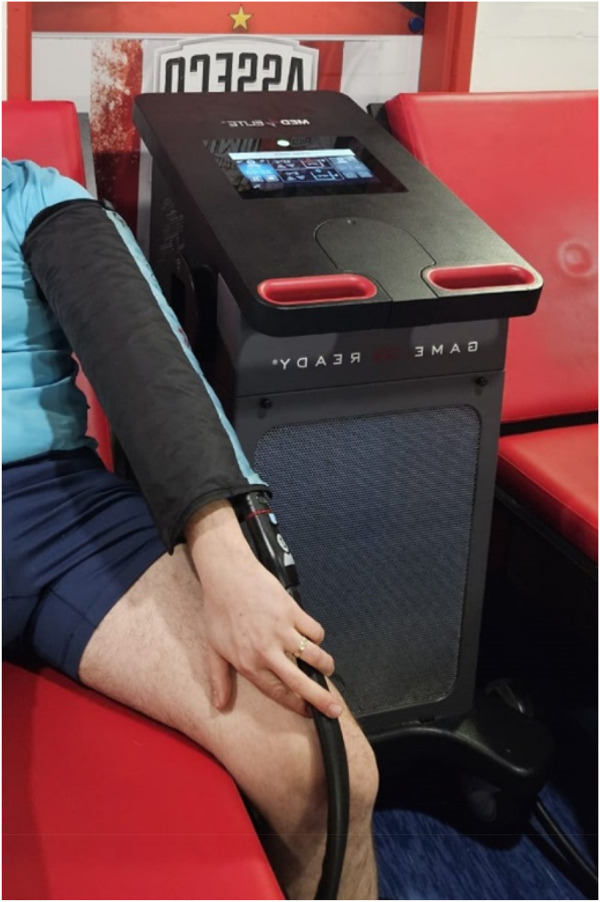
Game Ready contrast heat-cold compression therapy device.

In the experimental session (GRT), a Game Ready device (Avanos Medical, United States, 2020) was used with a cuff placed on the dominant upper limb (all participants were right-handed), which provided alternating stimulation for 1 minute with cold at a temperature of 3°C and a pressure of 75 mmHg (10 kPa), followed by 1 minute of heating at 45°C and compression at 15 mmHg (3.33 kPa). The total treatment time was 10 min. For the control group (GRS) receiving sham therapy, the same procedure was followed, consisting of 1 min of cold stimulus and 1 min of warm stimulus, over 10 min. The temperature was 15°C for a cold stimulus (lowest possible GR regulation) and a pressure of 15 mmHg (lowest possible GR regulation) and 36°C for a warm stimulus (neutral stimulus) at a pressure of 15 mmHg (Trybulski et al., 2024a). The least intense parameters were selected for the control group and the most intense ones for the experimental group to obtain the most significant effect, assuming that the body’s reaction is proportional to the applied stimulus, i.e., within the safe range for the participants (Figure 2). Seven days before the study, a familiarization session was performed, consisting of a 6-minute GR intervention with the same parameters as those used in the experimental session.

### 2.4 Assessment of tissue perfusion

**FIGURE 3:**
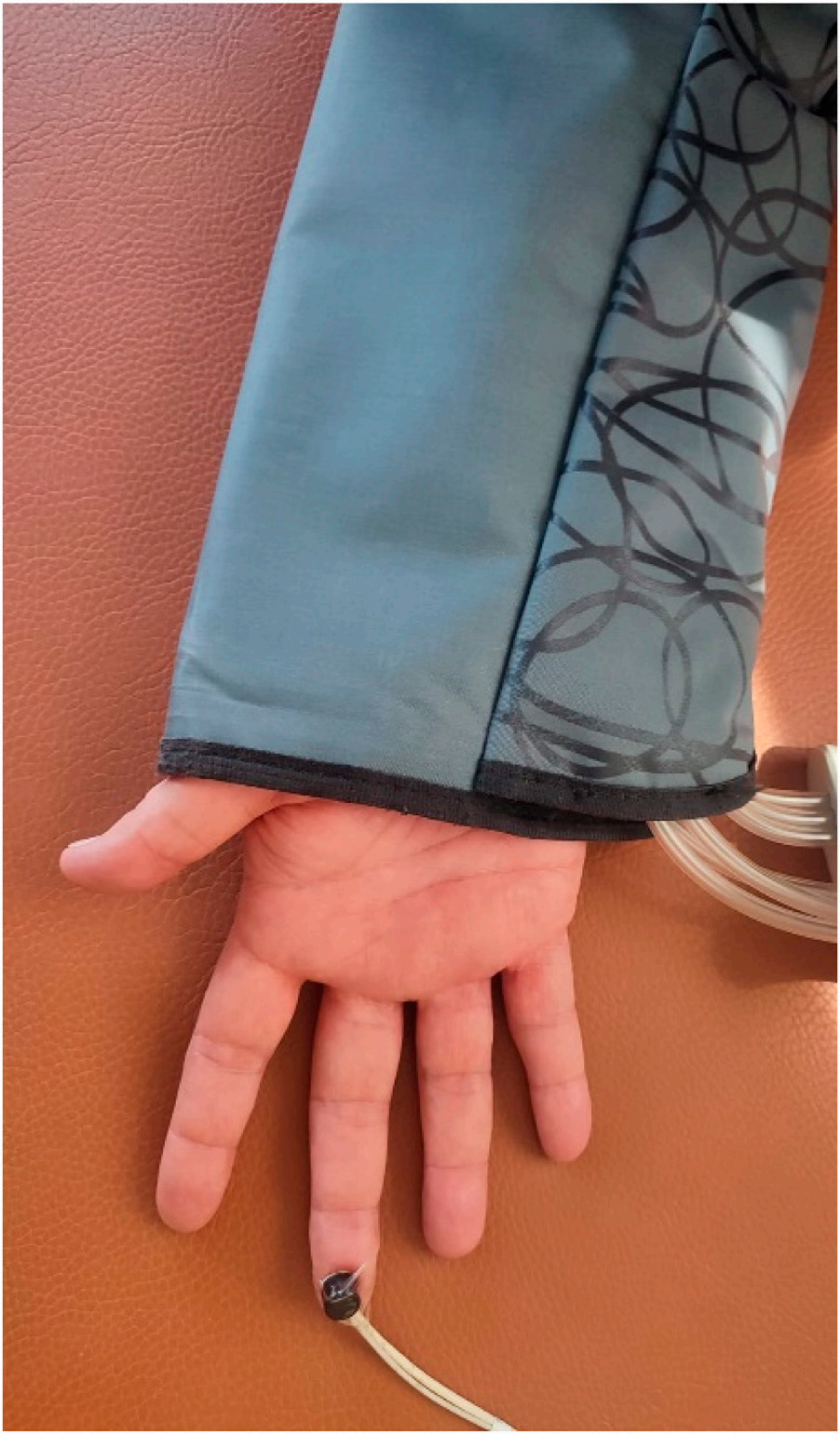
Placement of the LDF sensor on the middle finger.

Tissue perfusion was analyzed by laser Doppler flowmetry (LDF) using a Perimed device (Sweden, 2004). LDF is the gold standard in assessing microcirculatory responses, demonstrating high sensitivity and repeatability of measurements (Pavlov et al., 2017; Gemae et al., 2021). The wave reflected from erythrocytes was recorded at a skin tissue volume of (1 mm^3^) and a depth of 2.5 (mm) at a sampling rate of 32 [Hz]. The measurement was carried out using two contact laser probes on the examined area (tip of the middle finger of both hands) (Liana et al., 2009; Gemae et al., 2021). The laser-Doppler method uses monochromatic laser light with a narrow band from red to near-infrared (Babos et al., 2013). The device emits a beam of radiation deep into the tissue, which spreads within it. Photons encounter moving blood cells, changing their vibration frequency by the Doppler effect. Thus, flux is proportional to the velocity and concentration of erythrocytes in local tissue and is primarily impacted by cutaneous vasoconstriction and dilation changes (Saumet et al., 1988). The returning light is then analyzed using a photodetection system, and the camera generates a voltage directly proportional to the speed and number of moving blood cells in the examination area. The device records blood supply in the examined tissue area (Kvandal et al., 2006) (Figure 3). This study used well-established standardized measurements. The measurements were taken on the middle finger pad (Liana et al., 2009).

During the measurements, the participant was in a relaxed sitting position on an intravenous infusion chair, with the same elbow flexion angle (approximately 70°) as all participants (Trybulski et al., 2024b). Before starting the measurements, the participants rested for 15 min while waiting for the intervention (Liana et al., 2009). PU measurements were performed simultaneously on both forearms. The experimental intervention was used first, and due to the lack of hyperemic response on the contralateral side, measurements were abandoned during the sham session. The study was conducted at the Provita Medical Center between 8 a.m. and 12 p.m. The recorded air temperature was the same and amounted to 21°C. The following perfusion parameters were recorded (Liana et al., 2009; Gemae et al., 2021):

1. Rest flow (RF - [non-referent unit]), i.e., the average flow before the intervention, was performed within 2 min.
2. Therapeutic flow (TF - [min]), i.e., the average flow recorded during GR or sham therapy, were recorded for 10 min.
3. Time of recovery (TR - [min]), i.e., the time for perfusion to return to the resting value after the intervention.

First, measurements were performed for the intervention group on both sides. After the lack of effect on the contralateral side during the GR intervention, it was decided to discontinue recording contralateral perfusion during sham therapy. All measurements were performed by appropriately trained staff.

### 2.5 Statistical analysis

Means and standard deviations were used to represent the average and the typical spread of values for all analyzed data. The normality of the data distribution was verified using the Shapiro-Wilk test. A paired Student’s t-test was performed to detect statistical differences between trials. Effect sizes for pairwise comparisons were calculated using Cohen’s d and interpreted as trivial (<0.2), small (≥0.2), moderate (≥0.5), and large (≥0.8) (Cohen, 1992). Furthermore, the Minimal Detectable Change (MDC) was calculated to determine whether the observed effects are clinically significant (Table 2). *A priori* power analysis was conducted with the program G-Power (Düsseldorf University, Dusseldorf, Germany) (Faul et al., 2007). The difference between two dependent means with an effect size of at least 0.5, α = 0.05, and 1−β = 0.84 gave a statistical power of 85% and the total sample size of 30 subjects. The significance level was set to *p* < 0.05 for all analyses. All calculations were performed using TIBCO Statistica, v. 13.3.0. For graphical data presentation, the Durga library in the R (PBC, Boston, United States) programming environment was utilized (Khan and McLean, 2023).

**TABLE 2:** ICC, SEM, and MDC values for studied variable.

Variables	ICC_(1,k)_ [95 CI]	SEM	MDC_95_
PU	0.94 [0.81–0.97]	0.305	0.845

Note: ICC, intraclass correlation coefficient; SEM, standard error of measurement; MDC, minimal detectable change; PU, perfusion unit.

## 3 Results

**FIGURE 4:**
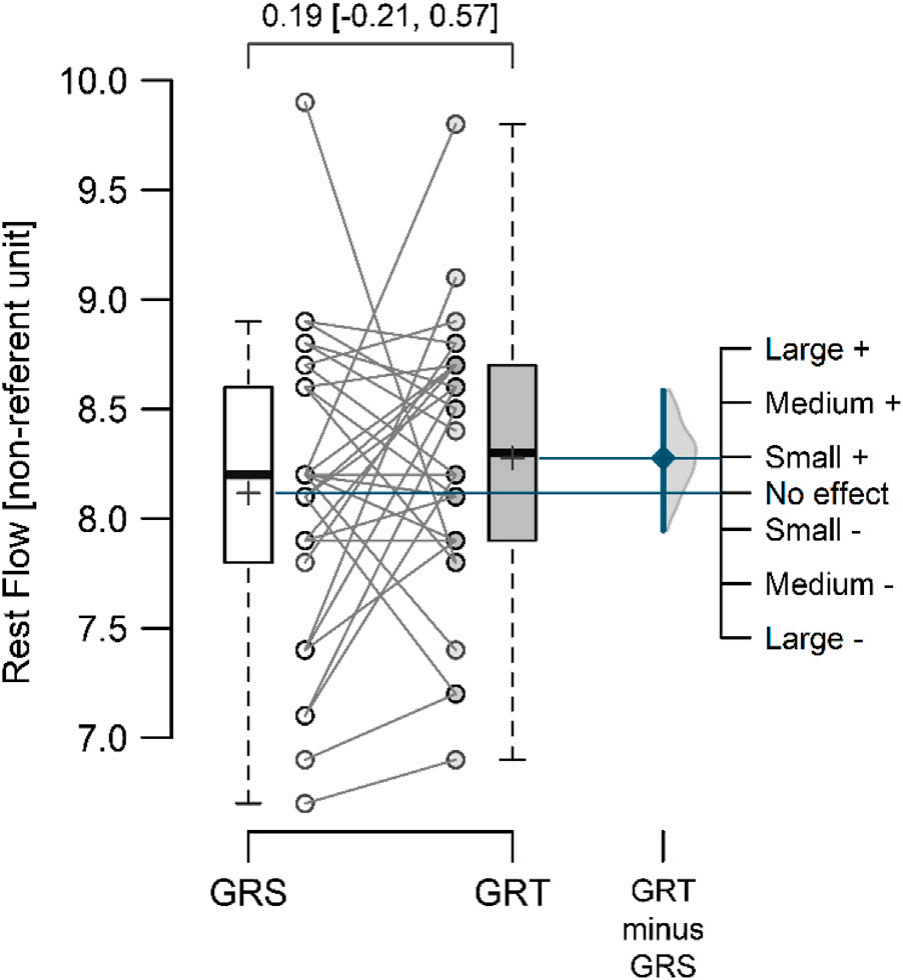
Mean resting perfusion flow values in the control (GRS, n = 30) and experimental (GRT, n = 30) groups, along with the standardized mean difference (Cohen’s d). Note: The values above the top brace express the effect size along with 95% confidence intervals.

**FIGURE 5:**
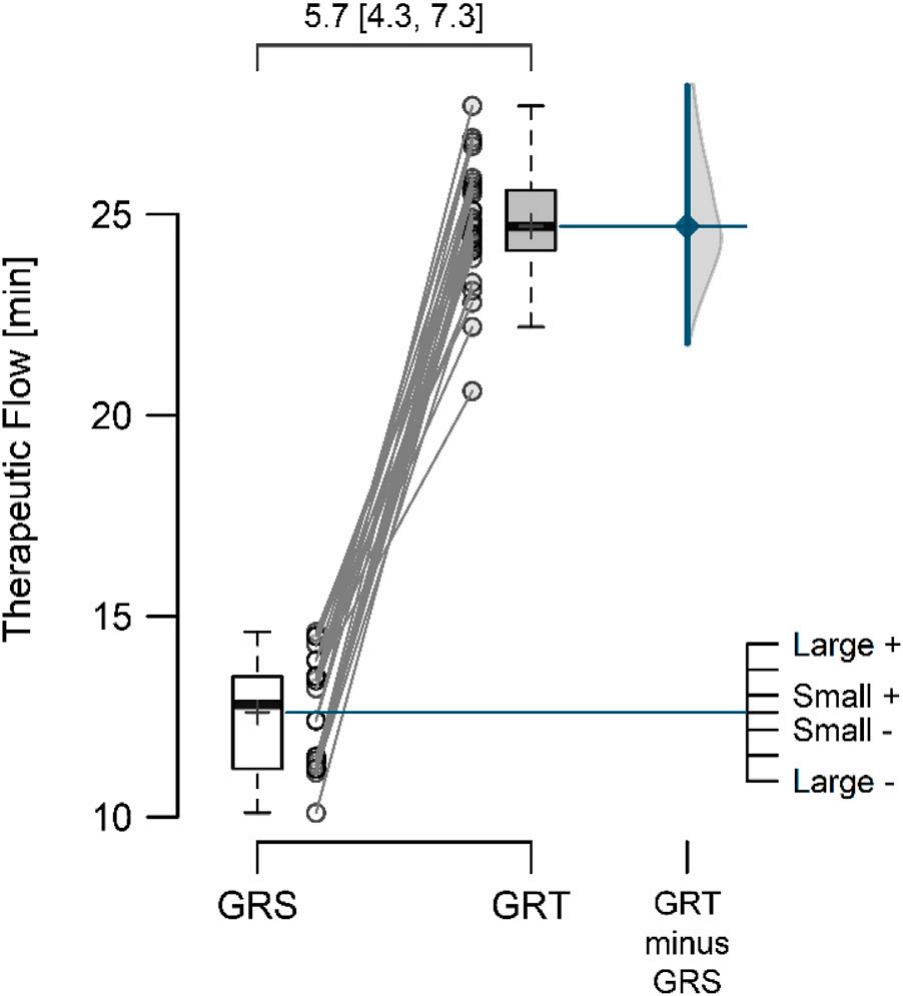
The mean perfusion therapeutic flow in the control (GRS, n = 30) and experimental (GRT, n = 30) groups, along with the standardized difference in means (Cohen’s d), is a significant aspect of our study. Note: The values above the top brace express the effect size along with 95% confidence intervals.

**FIGURE 6:**
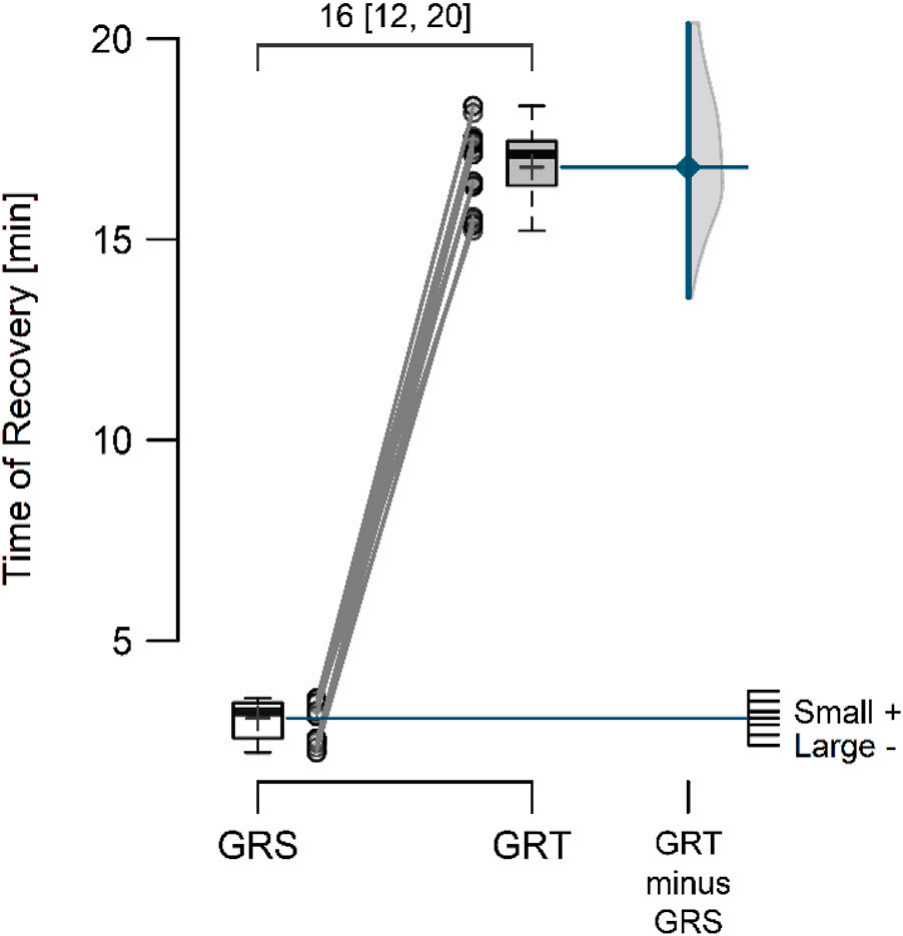
Time from the moment of cessation of contrast therapy to the moment of return of blood supply to the rest flow value in the control (GRS, n = 30) and experimental (GRT, n = 30) groups, along with the standardized difference in means (Cohen’s d). Note: The values above the top brace express the effect size along with 95% confidence intervals.

The mean resting perfusion values (Figure 4) in GRS and GRT did not differ significantly (8.12 ± 0.70 vs. 8.28 ± 0.62; *p* = 0.297; ES = 0.19 [small]; △ = 0.16 < MDC). Conversely, the mean perfusion during therapy (Figure 5) was significantly higher in GRT compared to GRS (24.70 ± 1.45 vs. 12.60 ± 1.37; *p* < 0.001; ES = 5.7 [large]; △ = 12.10 > MDC). Additionally, the time from cessation of contrast therapy to the return of blood flow to resting values (Figure 6) showed significantly higher values in GRT compared to GRS (3.07 ± 0.45 vs. 16.80 ± 0.91; *p* < 0.001; ES = 16.27 [large]).

When evaluating the contralateral effect on the mean resting perfusion value (Table 2), a statistically significant difference was observed between the right and left limbs (8.28 ± 0.62 vs. 7.74 ± 0.89; *p* = 0.014; ES = 0.48 [small]; △ = 0.53 < MDC). Significant differences were also observed in the mean perfusion during therapy between the right and left limbs (24.70 ± 1.45 vs. 7.66 ± 0.89; *p* < 0.001; ES = 13.34 [large]; △ = 17.05 > MDC). Importantly, no statistically significant difference was noted between the mean resting perfusion value (RF) and the mean perfusion value during therapy (TF) in the left limb (7.74 ± 0.89 vs. 7.66 ± 0.89; *p* = 0.284; ES = 0.20 [negligible]; △ = 0.09 < MDC), demonstrating the thoroughness of our research (Tables 3, 4).

**TABLE 3:** Comparison of the contralateral effect on perfusion between the right and left upper limbs.

Variable	Right - GRT	Left - GRS	△ (±95% CI)	p-val.	ES (±95% CI)
RF	8.28 ± 0.62	7.74 ± 0.89	0.53 (0.12; 0.95)	0.014	0.48 (0.10; 0.98)
TF	24.70 ± 1.45	7.66 ± 0.89	17.05 (16.57; 17.52)	<0.001	13.34 (10.93; 19.11)

Note: RF, mean resting perfusion value; TF, perfusion value during therapy; GRT, Game Ready Therapy; Left – contralateral limb; △ – differences between GRT and Left; p-val – *p*-value of the *t*-test.

**TABLE 4:** Comparison of the contralateral effect on perfusion on the left limb for resting and therapy perfusion values.

RF	TF	△ (±95% CI)	p-val	ES (±95% CI)
7.74 ± 0.89	7.66 ± 0.89	0.09 (−0.076; 0.249)	0.284	0.20 (−0.17; 0.57)

Note: RF, mean resting perfusion value; TF, perfusion value during therapy; △ – differences between RF and TF; p-val – *p*-value of the *t*-test.

## 4 Discussion

Our study aimed to evaluate changes in ipsilateral and contralateral perfusion after forearm contrast-compression therapy in MMA athletes. The results of this experiment confirm that there was an increase in hyperemic reactions on the ipsilateral side compared to sham therapy. However, no microvascular reactions were observed on the contralateral side. Significant changes concerned both therapeutic flows: i.e., the average flow recorded during GR therapy was recorded for 10 min, and the time perfusion returned to the resting value after the intervention. In this experiment, we used a 10-minute protocol based on previous observations, confirming that this is a sufficient time to obtain changes not only in perfusion but also in muscle biomechanical changes such as: muscle tension, muscle stiffness, and elasticity, as well as changes in muscle strength (Trybulski et al., 2024a).

Previous studies hypothesize that tissue blood supply reflects the microcirculation’s functional response (Joyner and Casey, 2014). These changes (flow-dependent dilation) determine the adaptive capacity of the vascular endothelium, which is reflected in post-exercise regeneration processes (Brunt et al., 2016). Heat or cold stress can result in different hemodynamic responses (Ogoh et al., 2013; Kalsi et al., 2017). Kim et al. confirmed increased blood flow measured by LDF, postulating that hyperemic responses facilitate recovery by promoting nutrient delivery (Kim et al., 2020a). In an animal experiment, Akasaki et al. demonstrated that repeated thermal therapy increased eNOS protein expression, blood flow, and capillary density in the ischemic hindlimb of mice (Akasaki et al., 2006). The endothelium plays a vital role in the mechanism of local autoregulation of microcirculation as a source of numerous mediators, the most potent of which are NO and prostacyclin, and the strongest vasoconstrictors are EDCF2 and endothelin (EDCF1) (Brunt and Minson, 2021).

Contrast therapy may affect the function of heat shock proteins (HSPs), which have a universal function in cellular homeostasis (Liu and Steinacker, 2001). HSP has been confirmed to be applicable in skeletal muscle, and its induction varies depending on the muscles’ histological and even functional characteristics (Xie et al., 2015). Local congestion may increase the release of adenosine-5′-triphosphate (ATP) from erythrocytes. Increasing intravascular ATP increases skin perfusion and equals or equals access due to blood stasis. ATP release from isolated erythrocytes was confirmed by heating and cooling (Kalsi et al., 2017).

The increase in microcirculatory response is described as an axonal reflex response that occurs locally upon applying variable stimuli (Abboud et al., 1976). Axonal reflex, i.e., the local neural reaction in response to contrast therapy, may also explain changes in microcirculation (Eriksson et al., 1986). Stimulation of pain or mechanical receptors can lead to the release of neuropeptide substances that can affect microcirculation (Horsman, 2006).

It is commonly accepted that contrast therapy using heat and cold in various forms increases muscle congestion, which reduces muscle tension and improves muscle stiffness and elasticity (Trybulski et al., 2024a). Although these mechanisms are unclear, non-myogenic regulation of muscle tone associated with increased perfusion is also accepted. The elevated cytosolic Ca^2+^ concentration in hypoxia conditions resulting from impaired perfusion causes muscle contraction by activating myosin light chain phosphorylation and subsequent actomyosin cross-bridging, increasing tension (Kim et al., 2020b). Activation of the capillary system, eliminating subclinical symptoms of tissue hypoxia, can, therefore, reduce muscle tension, which is important for preventing injuries (Huxel et al., 2008).

This study showed that ipsilateral contrast-compression therapy causes immediate microvascular effects, which disappear within 30 min after stimulation. The neurophysiological mechanisms underlying the increase in hyperemia in martial arts majors may be primarily attributable to their effects on the neuromuscular junction and pain modulation (Alexander et al., 2021). Contrast compression therapy enhances proprioceptive feedback and neuromuscular control through repetitive cycles of applying pressure, which can be used as mechanoreceptors in diseases and muscles (Kim and Choe, 2009). This process improves the use of motor units and slows down the system to a configuration that enables additional adequate muscle power (Donath and Faude, 2016).

Contralateral hyperemic reactions are important not only because they can influence an area that cannot be stimulated at a given time due to, for example, an acute injury or wound but also because of the programming of training in various vascular diseases, e.g., diabetic angiopathy (Balasubramanian et al., 2021). Although our observations do not confirm a contralateral effect, therapies such as compression (Martin et al., 2018) or contrast therapy may also affect microcirculation contralaterally (Ren et al., 2021). There is evidence that a reduction in blood flow in one area can lead to an increase in blood flow in contralateral areas, as a compensatory mechanism of the body (Nakayama et al., 1983). These mechanics in our body are so subject to automatic reactions that a rare phenomenon has been described in which the inflammatory response in one lower limb can lead to the “stealing” of blood from the healthy limb on the opposite side, resulting in ischemia of the normal limb in the absence of any actual stenosis of cryogenic vessels (Peer and Lovleen, 2024). This phenomenon can be explained by considering the hemodynamic homeostasis equation of fluid dynamics. The continuity equation applies to all fluids, regardless of their compressibility, whether Newtonian or non-Newtonian, and can be used to explain hemodynamic phenomena related to blood flow in the human body (Peer and Lovleen, 2024).

Concomitant contralateral responses in other studies are described as resulting from a centrally mediated reflex, possibly related to the bilaterality of the sensorimotor network induced by acute changes in the regional microcirculation (Rodrigues et al., 2020). The authors suggest that massage stimulation of one limb, regardless of time, appears to activate the same cardiovascular integration through a functional network that also includes the opposite counterpart to restore circulatory homeostasis on the side (Rodrigues et al., 2020).

Perfusion at the microvascular level involves the involvement of both local and central regulators as part of a complex vascular signaling system. One such regulatory response, critical in the lower limb, is the venoarterial reflex (VAR), which is particularly important in preventing edema or tissue damage and may also have a contralateral effect (Silva et al., 2018). Martin et al. showed that unilateral external pneumatic compression (EPC) could be an effective intervention to increase skin blood flow and peripheral vascular reactivity in the contralateral limb, which, according to the authors, is a reason to recommend the use of EPC on the healthy side in the treatment of chronic wounds on the contralateral side (Martin et al., 2018).

Opposite conclusions, partially consistent with ours, were reached by who, after a target filling pressure of 120 mmHg in patients after spinal cord injury, observed an increase in the reactivity of the posterior tibial artery in the treated (pressed) leg, but not in the untreated leg (Credeur et al., 2019).

## 5 Limitations of the study and future research directions

The scientific literature describes contralateral effects after the use of cold (Radecka et al., 2021) and warm (Ren et al., 2021) stimuli; however, our study is probably the first to analyze the contralateral effect in the microcirculation after the use of compression contrast therapy. Although this experimental study may provide valuable information on the effectiveness of contrast therapy in stimulating microcirculation in martial arts athletes, there are limitations to the study and opportunities for future research. First, the study focused only on short-term results, which warrants future investigation of the long-term effects of hyperemic reactions. Furthermore, although the lack of a contralateral perfusion effect was emphasized, further research is needed to elucidate the exact mechanisms underlying their effects on both limbs. Some limitation can be the fact that in this study only male participants were examined, so females should be included in future research. Future research should also examine individual differences in athletes’ responses to compression contrast therapy interventions, considering factors such as training experience or morphological and genetic predispositions.

## 6 Practical implications

The results of this study offer practical implications for optimizing ipsilateral hyperemic strategies that can potentially improve athletic performance. Athletes can accelerate muscle recovery, alleviate post-exercise fatigue, and improve overall athletic readiness. Trainers can strategically use contrast compression therapy in training programs or during competitions between bouts to mitigate the adverse effects of muscle fatigue. Unfortunately, the observed changes are local and short-lived. Based on our results, we cannot recommend using compression contrast therapy to influence adaptive changes in the contralateral microcirculation. More research is needed in this area.

## 7 Conclusion

Our crossover study showed a clinically significant ipsilateral hyperemic effect after applying compression contrast therapy to the forearm of MMA athletes compared to sham therapy. Unfortunately, this effect was only local and short-term (up to 20 min). The hyperemic reaction lasted longer (up to about 18 min) than the stimulation time itself (10 min), which can be used to program immediate regeneration effects in sports. The study did not confirm a contralateral hyperemic response. In this respect, we cannot recommend using this therapy to try to influence microcirculation in a place where the tissue cannot be stimulated directly, e.g., due to significant swelling or skin damage or the existence of difficult-to-heal wounds.

## Data Availability

The raw data supporting the conclusions of this article will be made available by the authors, without undue reservation.
